# Trajectory of gut microbiota before and after pediatric cardiopulmonary bypass surgery

**DOI:** 10.3389/fcimb.2024.1470925

**Published:** 2025-02-13

**Authors:** Xi Yin, Minhua Xiao, Jing Sun, Jinqing Feng, Shuliang Xia, Fengxiang Li, Xihong Liu, Jia Li

**Affiliations:** ^1^ Clinical Physiology Laboratory, Institute of Pediatrics, Guangzhou Women and Children’s Medical Center, Guangzhou, China; ^2^ Department of Nutrition, Guangzhou Women and Children’s Medical Center, Guangzhou, China; ^3^ Heart Center, Guangzhou Women and Children’s Medical Center, Guangzhou, China

**Keywords:** congenital heart disease, ventricular septal defect, tetralogy of Fallot, cardiopulmonary bypass, children, gut microbiota

## Abstract

**Background:**

Varied congenital heart disease (CHD) may induce gut microbiota dysbiosis due to intestinal hypoperfusion or/and hypoxemia. Microbiota dysbiosis has been found in preoperative infants and cardiopulmonary bypass (CPB) exacerbated it further. However, the trajectory of gut microbiota from pre- to early post-CPB and one-year later remains unexplored. We examined this trajectory in the two most common CHDs, i.e., left-to-right shunt (ventricular septal defect, VSD) vs. right-to-left shunt (tetralogy of Fallot, TOF).

**Methods:**

We enrolled 13 infants with VSD and 11 with TOF, and collected fecal samples at pre- and early post-CPB. 10 and 12 age- and gender-matched healthy control infants were enrolled respectively. We also enrolled 13 and 9 gender- and CHD diagnosis- and operation-matched one-year post-CPB patients, and 8 age- and gender-matched healthy control children. 16S rRNA sequencing of fecal samples were performed.

**Results:**

Compared to the control groups, both VSD and TOF pre-CPB groups had significantly increased Enterobacteriaceae and *Shigella*, and decreased *Bifidobacterium* (*Ps ≤* 0.049). No significant change in microbial community diversity was observed between pre- and early post-CPB periods (*Ps*≥0.227). Compared with early post-CPB, one-year post-CPB groups had significantly increased short-chain fatty acids-producing microbes (*Ps* ≤ 0.025), and their microbial communities were close to that of the control group (*Ps*≥0.102). There was no significant difference in microbial communities between VSD and TOF groups in any of 3 periods (*Ps*≥0.055).

**Conclusion:**

In children with VSD or TOF, gut microbiota dysbiosis existed preoperatively and were not significantly altered by CPB. One-year post-CPB, microbiota significantly improved towards normal. Similar microbial communities were found between children with VSD and TOF throughout the perioperative and long-term postoperative periods.

## Introduction

In healthy conditions, gut microbiota has symbiotic interactions with the host, and the community is typically dominated by bacteria (Clemente et al., 2012; Hou et al., 2022). Intestinal bacteria serve diverse functions, such as food fermentation and defense against pathogens which underlines profound implications on human health (Hou et al., 2022). Disruption of gut microbiota has been found in the occurrence and progression of diseases, such as critical illness and malnutrition (Wijeyesekera et al., 2019; Patterson et al., 2022).

Patients with congenital heart disease (CHD) are at risk of intestinal barrier dysfunction and gut dysbiosis as a result of intestinal hypoperfusion caused by reduced systemic blood flow in the left to right shunt type or intestinal tissue hypoxia caused by hypoxemia in the right to left shunt type or both in more complex CHD (Feng et al., 2021). It has been found that disrupted microbiota existed preoperatively and difference exhibited between patients with mild and non-cyanotic CHD (e.g., ventricular septal defect, VSD) and those with severe and cyanotic CHD (e.g., tetralogy of Fallot (TOF), transposition of the great arteries, double outlet right ventricle, etc.) (Liu et al., 2022; Salomon et al., 2021; Huang et al., 2022; Xing et al., 2018). Studies showed that preoperative patients with these complex and cyanotic CHD had different microbial composition with decreased proportion of *Lactobacilli* compared to patients with non-cyanotic CHD (Xing et al., 2018) and that dysbacteriosis was related to inflammatory status and postoperative adverse outcomes (Huang et al., 2022). Gut microbiota in infants with left-to-right shunt CHD and heart failure was featured by increased pathogenic bacteria, such as *Enterococcus* and *Shigella*, while beneficial ones, such as *Bifidobacterium*, were decreased (Zhang et al., 2023).

Cardiopulmonary bypass (CPB) induces intense systemic inflammatory response, ischemia-reperfusion injury and impaired systemic hemodynamics. Thus, CPB may exacerbate intestinal barrier dysfunction and microbiota dysbiosis (Salomon et al., 2021), which inversely intensifies systemic inflammatory response (Feng et al., 2021; Salomon et al., 2021; Pathan et al., 2011). It has been found that the dysbiosis was deteriorated and fecal concentrations of eicosane compounds associated with pro-inflammatory signals were elevated after CPB (Salomon et al., 2021).

During the early years of life, gut microbiota shows strong association with the growth and development of children (Carlson et al., 2018; Tsukuda et al., 2021). We have previously reported that children with CHD remained underdeveloped even until 6 months after CPB (Shi et al., 2021). But the trajectory of gut microbiota from pre- to early and long term post-CPB remains unexplored. Therefore, characterizing their features may provide insights to understand the mechanisms of poor growth and development of children with CHD before and after CPB.

In this study, we selected more uniform and most common types of left to right shunt and right to left shunt CHD, that is, VSD and TOF, and examined the characteristic and trajectory of gut microbiota from pre- to early and long-term post-CPB periods. We hypothesized that disrupted gut microbiota would be characteristically different before CPB between VSD and TOF patients, and would be further exacerbated early after CPB. There would be gradual recovery by one year after CPB.

## Materials and methods

### Subjects and samples collection

The study was approved by the Research Ethics Committee of Guangzhou Women and Children’s Medical Center (NO.46201). Informed consents were obtained from the parents.

**Figure 1:**
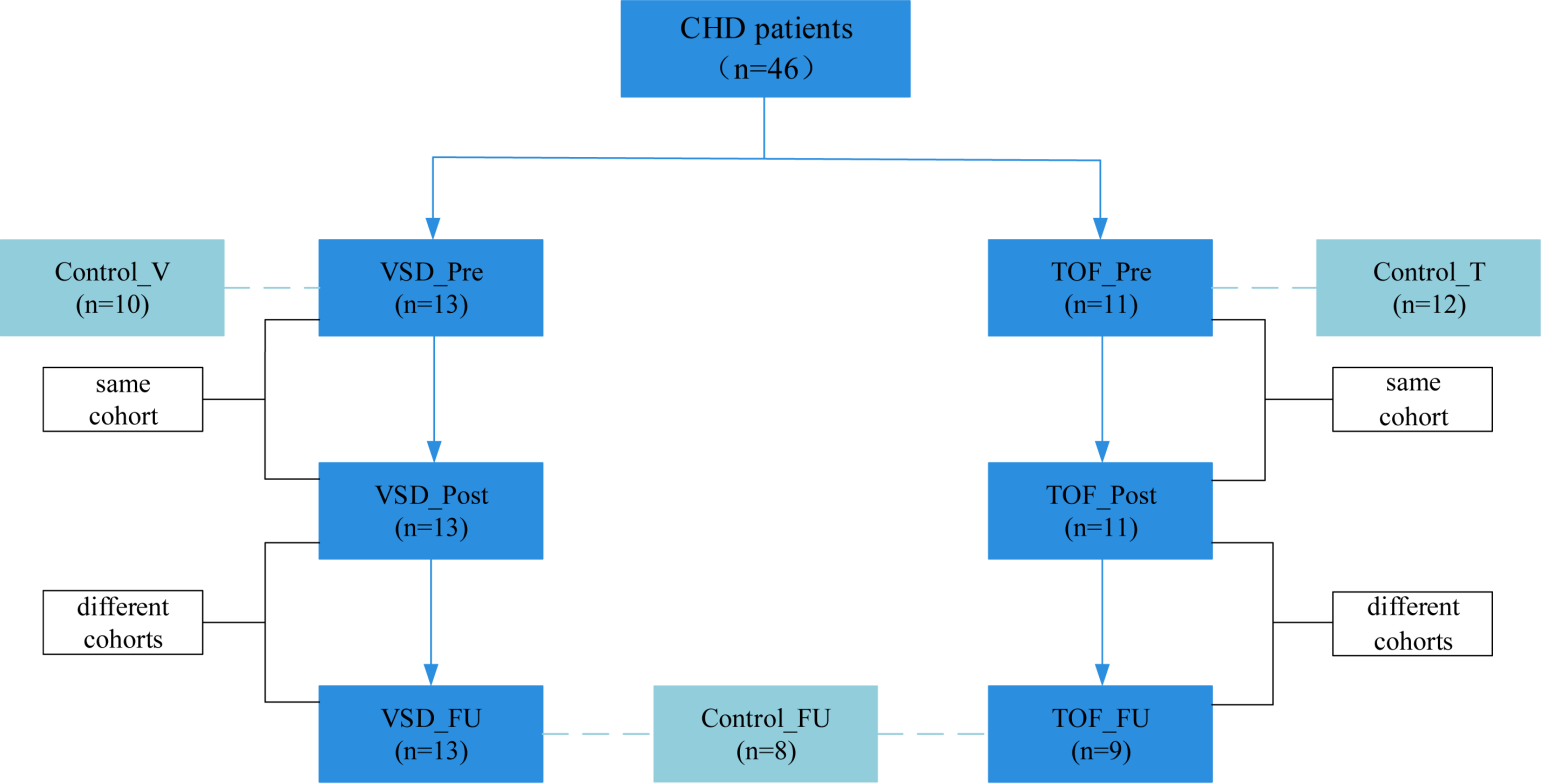
Enrollment process. CHD, congenital heart disease; Control_V, control group corresponding to ventricular septal defect; VSD_Pre, preoperative group with ventricular septal defect; VSD_Post, early postoperative group with ventricular septal defect; VSD_FU, one-year postoperative group with ventricular septal defect; Control_T, control group corresponding to tetralogy of Fallot; TOF_Pre, preoperative group with tetralogy of Fallot; TOF_Post, early postoperative group with tetralogy of Fallot; TOF_FU, one-year postoperative group with tetralogy of Fallot; Control_FU, control group corresponding to one-year postoperative groups.

*CHD groups.* We enrolled infants with VSD (n=13, aged 3.7 ± 1.7 months) and TOF (n=11, aged 4.8 ± 2.4 months) who were scheduled for CPB from January 2021 to January 2022. The pre-CPB (VSD_Pre and TOF_Pre) and post-CPB (VSD_Post and TOF_Post) subjects were the same cohort with repeated samplings. During the same period, we also enrolled gender- and CHD diagnosis- and operation-matched groups of children at about 1 year post-CPB (VSD_FU and TOF_FU, n=13 and 9, aged 20.9 ± 4.4 months and 23.2 ± 4.3 months, respectively) (**Figure 1**). Exclusion criteria included gestational age<36 weeks (n=5), having gastrointestinal diseases (n=3), failure to obtain samples (n=3), and antibiotics or probiotics treatment within the past month (n=2) It should be mentioned that these patients routinely received prophylactic antibiotics prior to CPB.

*Control groups.* Since gut microbiota has been shown to have a distinctive temporal characteristic during the first year of life (Roswall et al., 2021; Yassour et al., 2016; de Muinck and Trosvik, 2018), we enrolled two separate control groups to match age with preoperative groups better, respectively. Healthy infants and children consulting in the outpatient clinic of the Department of Clinical Nutrition were screened and recruited, and were gender- and age-matched with pre-CPB (Control_V and Control_T, n=10 and 12, aged 3.5 ± 1.5 months and 5.3 ± 2.6 months, respectively) and 1 year post-CPB subjects (Control_FU, n=8, aged 24.0 ± 6.1 months) (**Figure 1**). Exclusion criteria included gestational age<36 weeks (n=3), having gastrointestinal diseases (n=3), antibiotics or probiotics treatment within the past month (n=3), and diagnosed with malnutrition (n=2) and milk allergy (n=3).

Fecal samples were obtained from diapers at the corresponding times described above and stored at -80°C within 2 hours.

### Demographic and clinical data collection

Demographic data (including age, weight, gender, gestational age, delivery mode, and feeding mode) and clinical data (including prophylactic antibiotic use prior to CPB, durations of CPB, aortic cross-clamping, postoperative mechanical ventilation, CICU and hospital stay) were recorded.

### 16S rRNA sequencing and analysis

The 16S rRNA sequencing analysis was performed by Metabo-Profile Biotechnology (Shanghai, China) Co.,Ltd. Total genomic DNA was extracted using the Mag-Bind Soil DNA Kit (Omega Bio-Tek, USA). The 16S rRNA genes V3-V4 regions were amplified using primers 338F (ACTCCTACGGGAGGCAGCA) and 806R (GGACTACHVGGGTWTCTAAT). Amplicons were quantified using the Quant-iT PicoGreen dsDNA Assay Kit (Invitrogen, Carlsbad, CA, USA) and sequencing was performed using the Illlumina NovaSeq platform (Illumina, San Diego, California, USA).

Microbiome bioinformatics were performed with QIIME2 (version 2019.4). Sequences were quality filtered, denoised, merged and chimera removed using DADA2 plugin. Taxonomy was assigned to amplicon sequence variants (ASVs) using the Naive Bayes taxonomy classifier against the Greengenes Release 13.8 Database. Alpha diversity (Chao1 and Shannon indices) was calculated using the ASV table. Beta diversity was visualized via principal-coordinate analysis based on weighted UniFrac distance. Permutational multivariate analysis of variance assess the significance of differences in beta diversity between groups. The relative abundances of abundant microbiota at genus level were compared between two groups. If two groups were matched, matched samples t-test or Wilcoxon signed-rank were used to compare it based on whether the difference values were outliers, or not, and the normality of them. And if the groups were not matched, independent samples t-test or Mann-Whitney U test were used to compare it based on the normality of data and homogeneity of variances. When microbial diversity and the relative abundances of abundant genera were significantly different between two groups, Linear discriminant analysis effect size (LEfSe) was performed to identify the characteristic taxa between them further (linear discriminant analysis score2.0, *P*0.05).

### Statistical analysis

Shapiro-Wilk test and Levene’s test were used to test the normality of data and homogeneity of variances. Normally distributed continuous variables were tested using independent samples t-test and presented as mean ± SD. Non-normally distributed continuous variables were presented as median (interquartile range) and tested using Mann-Whitney U test. The numbers and percentages were reported for categorical variables, and Fisher’s Exact test was used. The data were analyzed using SPSS version 20.0 (IBM, Armonk, USA). *P*-value0.05 was considered statistically significant.

## Results

### Demographic and clinical characteristics

All the patients had smooth recovery after CPB. There were no major complications. Prophylactic antibiotics cefazolin sodium was routinely administered prior to CPB. There were 7 and 8 patients of VSD_Post and TOF_Post groups, respectively, who were treated with ceftazidime due to suspected infection postoperatively before sampling.

There was no significant difference in demographics between Control_V and Control_T groups, between VSD_Pre and Control_V groups and between TOF_Pre and Control_T groups, except for infants in VSD_Pre group who some were primarily delivered vaginally compared to Control_V group (40.0% vs. 84.6%, *P*=0.039). Compared with VSD_Pre group, TOF_Pre group had higher weight (5.2±1.2 vs. 6.2±1.1kg, *P*=0.049). VSD_Pre and TOF_Pre groups samples were collected at 1.3±0.5 and 2.5±1.7 days prior to CPB (*P*=0.119) (**Table 1**).

**Table 1:** Demographic and clinical data of preoperative groups and control groups.

	Control_V(n=10)	VSD_Pre(n=13)	Control_T(n=12)	TOF_Pre(n=11)	*p*-value (Control_V vs. Control_T)	*p*-value (Control_V vs. VSD_Pre)	*p*-value (Control_T vs. TOF_Pre)	*p*-value (VSD_Pre vs. TOF_Pre)
Age (months)	3.5 ± 1.5	3.7 ± 1.7	5.3 ± 2.6	4.8 ± 2.4	0.066	0.725	0.674	0.190
Male (n%)*	6 (60.0%)	7 (53.8%)	8 (66.7%)	5 (45.5%)	>0.999	>0.999	0.414	>0.999
Birth weight (Kg)	3.4 ± 0.6	3.2 ± 0.6	3.3 ± 0.6	3.0 ± 0.4	0.539	0.327	0.051	0.304
Gestational age (weeks)	38.9 ± 1.2	38.9 ± 1.2	39.0 (39.0-38.0)	38.0 (39.0-38.0)	0.767	0.964	0.169	0.068
Weight (Kg)	6.3 ± 1.1	5.2 ± 1.2	6.9 ± 1.1	6.2 ± 1.1	0.180	0.057	0.152	0.049
Vaginal delivery (n%)	4 (40.0%)	11 (84.6%)	6 (50.0%)	7 (63.6%)	0.691	0.039	0.680	0.357
Feeding mode (n%)								
Breastfed	5 (50.0%)	5 (38.5%)	7 (58.3%)	4 (36.4%)	>0.999	0.685	0.414	>0.999
Mixed-fed	5 (50.0%)	5 (38.5%)	5 (41.7%)	7 (63.6%)	>0.999	0.685	0.414	0.414
Exclusively formula-fed	0	3 (23.1%)	0	0	—	—	—	—
Complementary feeding (n%)	0	3 (23.1%)	3 (25.0%)	4 (36.4%)	—	—	0.667	0.659
Preoperative sampling times (days)	—	1.3 ± 0.5	—	2.5 ± 1.7	—	—	—	0.119

Values are mean ± SD or median (interquartile range) or n (%).

*The number of males is after excluding females; the male % indicates the percentage of males in the respective groups including females.

Control_V, control group corresponding to ventricular septal defect; VSD_Pre, preoperative group with ventricular septal defect; Control_T, control group corresponding to tetralogy of Fallot; TOF_Pre, preoperative group with tetralogy of Fallot.

The duration of CPB [75 (84-64) vs. 115 (126-96) min, *P*<0.001] and aortic cross-clamping [54 (61-42) vs. 66 (82-62) min, *P*=0.003], and hospital length of stay (9.9±2.2 vs. 14.0±3.2 days, *P*=0.001) were longer in TOF_Post group than in VSD_Post group. VSD_Post and TOF_Post groups samples were collected at 4.6±1.3 days and 6.0±2.0 days post-CPB (*P*=0.093) (**Table 2**).

**Table 2:** Clinical data of early postoperative groups.

	VSD_Post (n=13)	TOF_Post (n=11)	*p*-value
Surgical time (min)	133 ± 25	198 ± 67	<0.001
CPB time (min)	75 (84-64)	115 (126-96)	<0.001
Aortic cross-clamping time (min)	54 (61-42)	66 (82-62)	0.003
Ventilator time (days)	1.0 (2.5-0.5)	1.0 (2.0-0.5)	>0.999
Antibiotics time (days)	1.5 ± 2.0	3.2 ± 2.4	0.119
Early postoperative sampling time (days)	4.6 ± 1.3	6.0 ± 2.0	0.093
CICU LOS (days)	2.9 ± 1.9	3.9 ± 1.5	0.119
Hospital LOS (days)	9.9 ± 2.2	14.0 ± 3.2	0.001

Values are mean ± SD or median (interquartile range).

VSD_Post, early postoperative group with ventricular septal defect; TOF_Post, early postoperative group with tetralogy of Fallot; CPB, cardiopulmonary bypass; CICU, cardiac intensive care unit; LOS, length of stay.

Compared with Control_FU group, VSD_FU group had lower weight (11.9±1.9 vs. 9.9±2.1 kg, *P*=0.040). No significant difference in demographic data was observed between Control_FU and TOF_FU groups, nor between VSD_FU and TOF_FU groups (*Ps*≥0.057). VSD_FU and TOF_FU groups samples were collected at 15.8 ± 4.6 months and 16.9 ± 4.0 months post-CPB (*P*=0.601) (**Table 3**).

**Table 3:** Demographic and clinical data of long-term postoperative groups and control group.

	Control_FU (n=8)	VSD_FU (n=13)	TOF_FU(n=9)	*p*-value (Control_FU vs. VSD_FU)	*p*-value (Control_FU vs. TOF_FU)	*p*-value (VSD_FU vs. TOF_FU)
Age (months)	24.0 ± 6.1	20.9 ± 4.4	23.2 ± 4.3	0.197	0.541	0.292
Male (n%)*	2 (25.0%)	5 (38.5%)	7 (77.8%)	0.656	0.057	0.099
Weight (Kg)	11.9 ± 1.9	9.9 ± 2.1	10.4 ± 1.4	0.040	0.074	0.572
Long-term postoperative sampling time (months)	—	15.8 ± 4.6	16.9 ± 4.0	—	—	0.601

Values are mean ± SD or n (%).

*The number of males is after excluding females; the male % indicates the percentage of males in the respective groups including females.

Control_FU, control group corresponding to one-year postoperative groups; VSD_FU, one-year postoperative group with ventricular septal defect; TOF_FU, one-year postoperative group with tetralogy of Fallot.

### Microbial characteristics before CPB, early and one-year post-CPB

**Figure 2:**
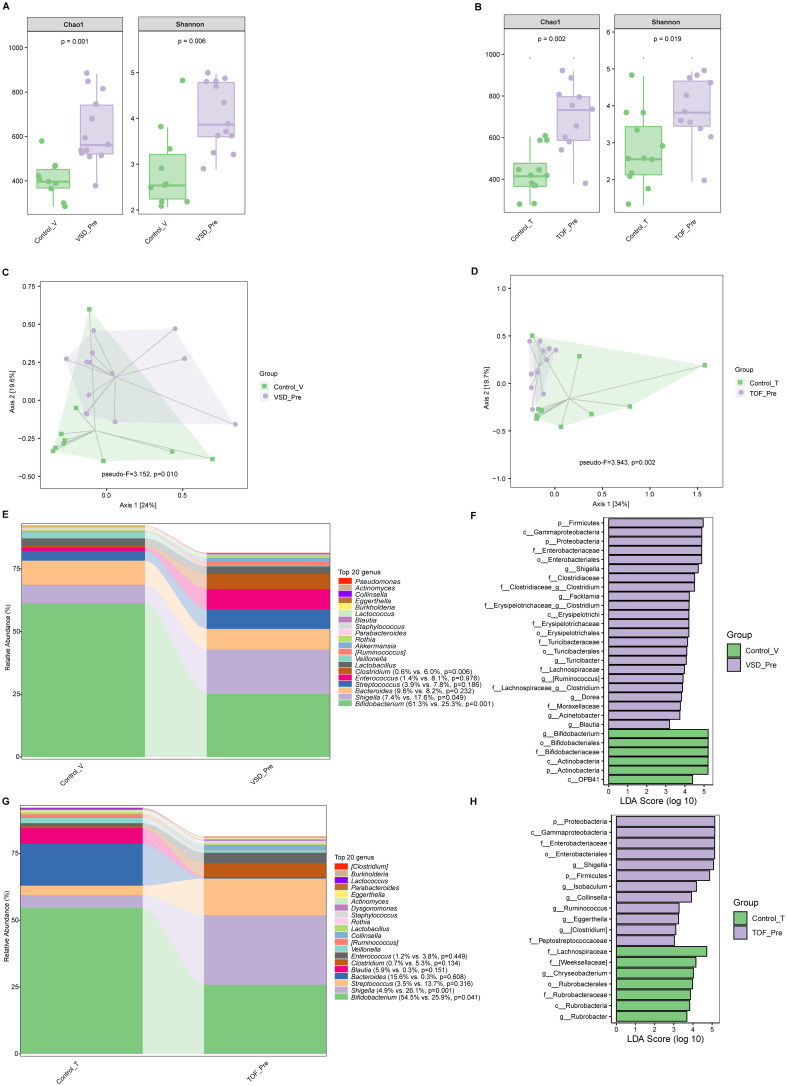
The comparison of alpha diversity between Control_V and VSD_Pre groups **(A)**, and between Control_T and TOF_Pre groups **(B)**. Principal-coordinate analysis based on weighted UniFrac distance between Control_V and VSD_Pre groups **(C)**, and between Control_T and TOF_Pre groups **(D)**. The top 20 relative abundances of bacteria at genus level in Control_V and VSD_Pre groups **(E)**, and LEfSe analysis between them **(F)**. The top 20 relative abundances of bacteria at genus level in Control_T and TOF_Pre groups **(G)**, and LEfSe analysis between them **(H)**. Control_V, control group corresponding to ventricular septal defect; VSD_Pre, preoperative group with ventricular septal defect; Control_T, control group corresponding to tetralogy of Fallot; TOF_Pre, preoperative group with tetralogy of Fallot.

Compared with Control_V and Control_T groups respectively, both VSD_Pre and TOF_Pre groups had significantly increased alpha diversity (*Ps* ≤0.019) (**Figures 2A, B**). There were significant differences in beta diversity between pre-CPB and corresponding control groups (*Ps* ≤0.010), respectively (**Figures 2C, D**). Both VSD_Pre and TOF_Pre groups showed a significantly decreased relative abundance of *Bifidobacterium*, and were enriched with *Gammaproteobacteria, Enterobacteriaceae*, and *Shigella* compared to corresponding control groups (*Ps* ≤ 0.049) (**Figures 2E–H**). VSD_Pre group also presented the higher relative abundance of *Clostridium* compared to Control_V group (*P*=0.006) (**Figure 2E**). 

**Figure 3:**
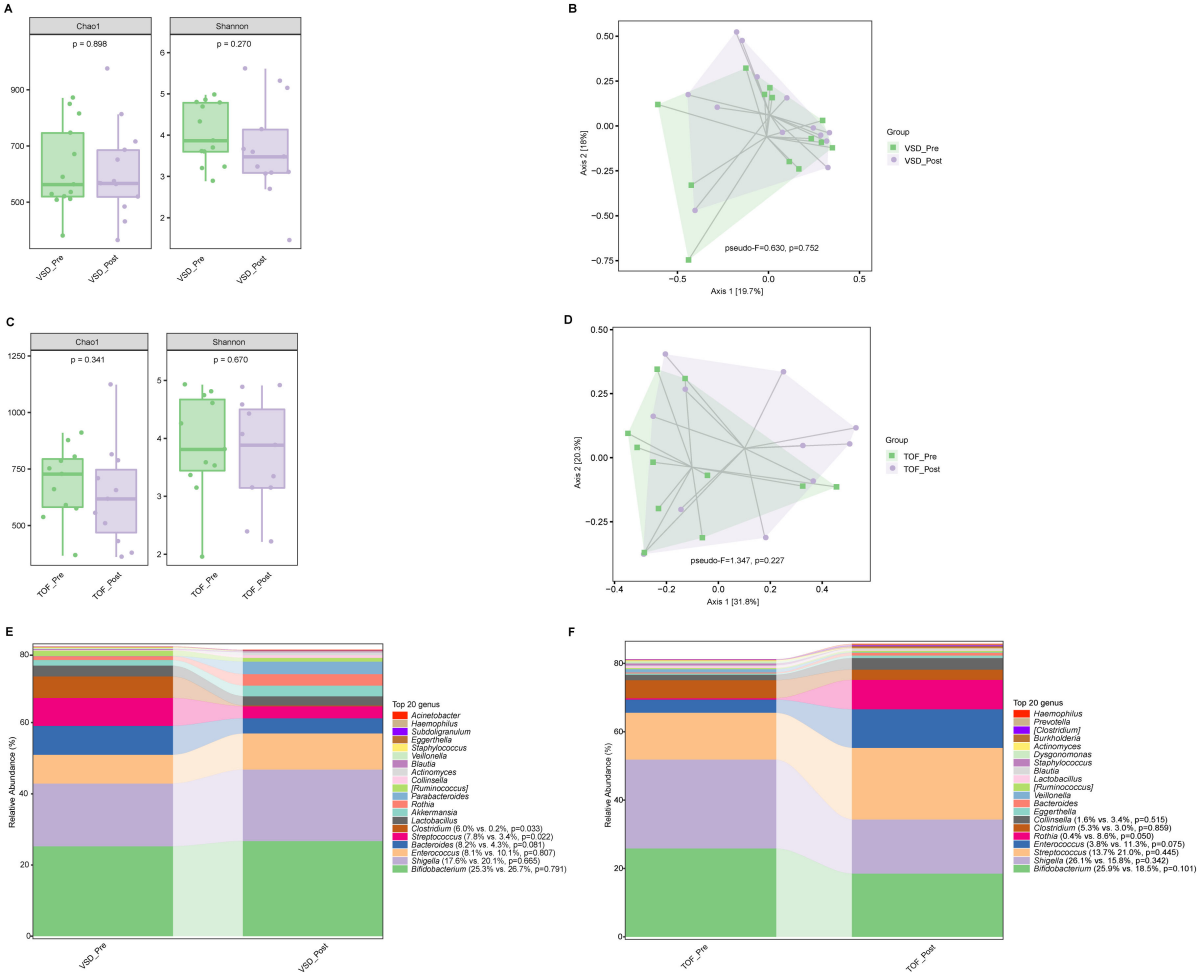
The comparisons of alpha diversity **(A)** and principal-coordinate analysis based on weighted UniFrac distance **(B)** between VSD_Pre and VSD_Post groups. The comparisons of alpha diversity **(C)** and principal-coordinate analysis based on weighted UniFrac distance **(D)** between TOF_Pre and TOF_Post groups. The top 20 relative abundances of bacteria at genus level in VSD_Pre and VSD_Post groups **(E)**, and in TOF_Pre and TOF_Post groups **(F)**. VSD_Pre, preoperative group with ventricular septal defect; VSD_Post, early postoperative group with ventricular septal defect; TOF_Pre, preoperative group with tetralogy of Fallot; TOF_Post, early postoperative group with tetralogy of Fallot.

There was no significant difference in alpha diversity and beta diversity between VSD_Pre and VSD_Post groups (*Ps*≥0.270) (**Figures 3A, B**), nor between TOF_Pre and TOF_Post groups (*Ps* ≥ 0.227) (**Figures 3C, D**). The relative abundances of abundant genera were not significantly different between pre-CPB and post-CPB groups (*Ps*≥ 0.050), except for *Streptococcus* (P=0.022) and *Clostridium* (*P*=0.033) being decreased in VSD_Post group compared to VSD_Pre group (**Figures 3E, F**).

**Figure 4:**
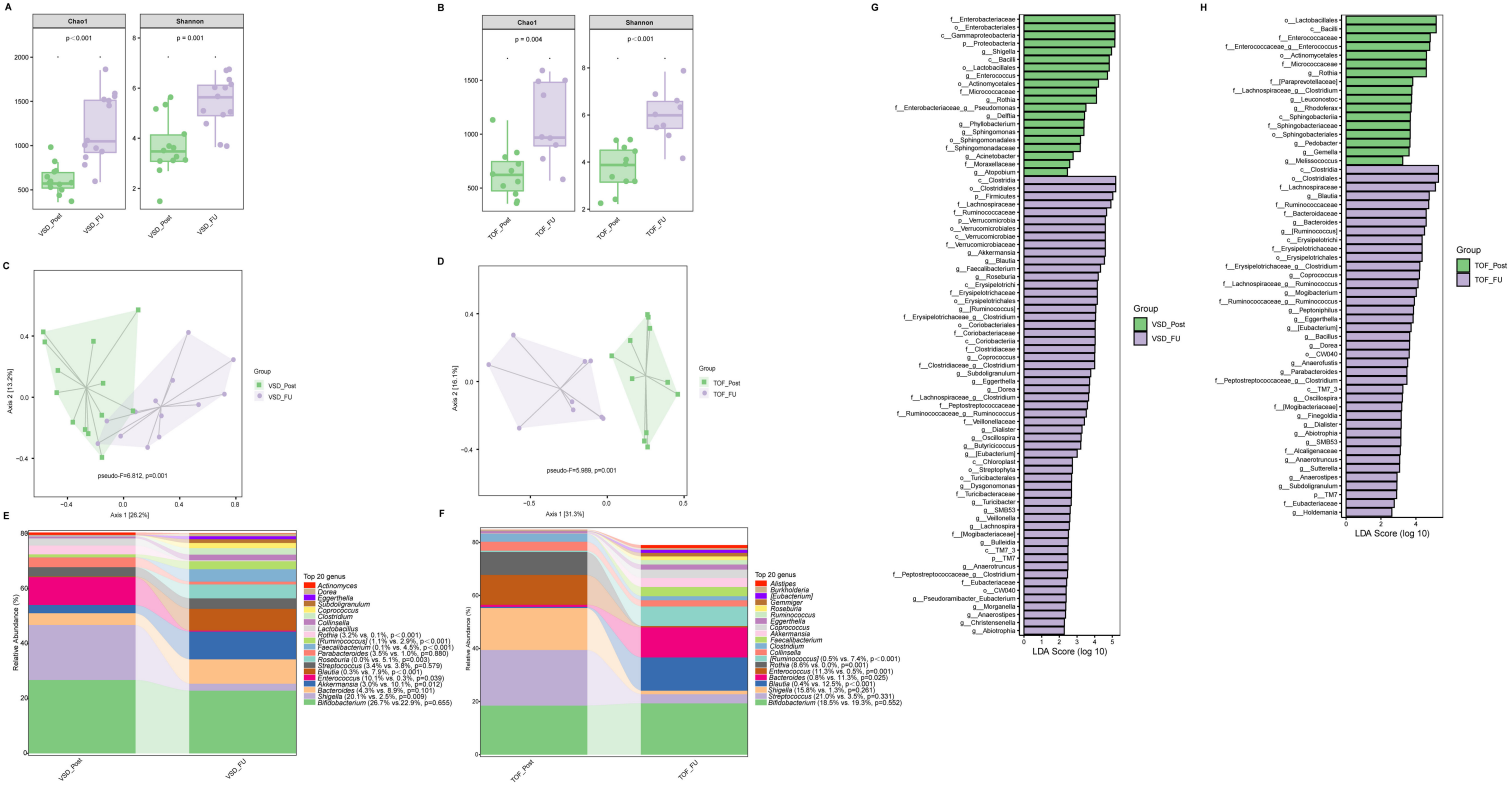
The comparison of alpha diversity between VSD_Post and VSD_FU groups **(A)**, and between TOF_Post and TOF_FU groups **(B)**. Principal-coordinate analysis based on weighted UniFrac distance between VSD_Post and VSD_FU groups **(C)**, and between TOF_Post and TOF_FU groups **(D)**. The top 20 relative abundances of bacteria at genus level in VSD_Post and VSD_FU groups **(E)**, and in TOF_Post and TOF_FU groups **(F)**. LEfSe analysis between VSD_Post and VSD_FU groups **(G)**, and between TOF_Post and TOF_FU groups **(H)**. VSD_Post, early postoperative group with ventricular septal defect; VSD_FU, one-year postoperative group with ventricular septal defect; TOF_Post, early postoperative group with tetralogy of Fallot; TOF_FU, one-year postoperative group with tetralogy of Fallot.

Compared with VSD_Post and TOF_Post groups respectively, VSD_FU and TOF_FU had significantly increased alpha diversity (*Ps* ≤ 0.004) (**Figures 4A, B**) and significant differences in beta diversity (*Ps*=0.001) (**Figures 4C, D**), and they were characterized by a significant dominance of short-chain fatty acids (SCFAs)-producing bacteria and decrease of *Enterococcus* and *Rothia* (*Ps* ≤ 0.039) (**Figures 4E–H**). Specifically, in VSD_FU group, the relative abundances of SCFAs-producing taxa including *Blautia, [Ruminococcus], Coprococcus*, *Dorea, Oscillospira, Akkermansia, Roseburia, Faecalibacterium*, and *Butyricicoccus* were significantly higher than those of VSD_Post group, and the proportions of Enterobacteriaceae and *Shigella* were lower (**Figure 4G**). The relative abundances of SCFAs-producing taxa including *Bacteroides, Blautia, [Ruminococcus], Coprococcus*, *Dorea*, and *Oscillospira* were significantly higher in TOF_FU group compared with in TOF_Post group (**Figure 4H**).

**Figure 5:**
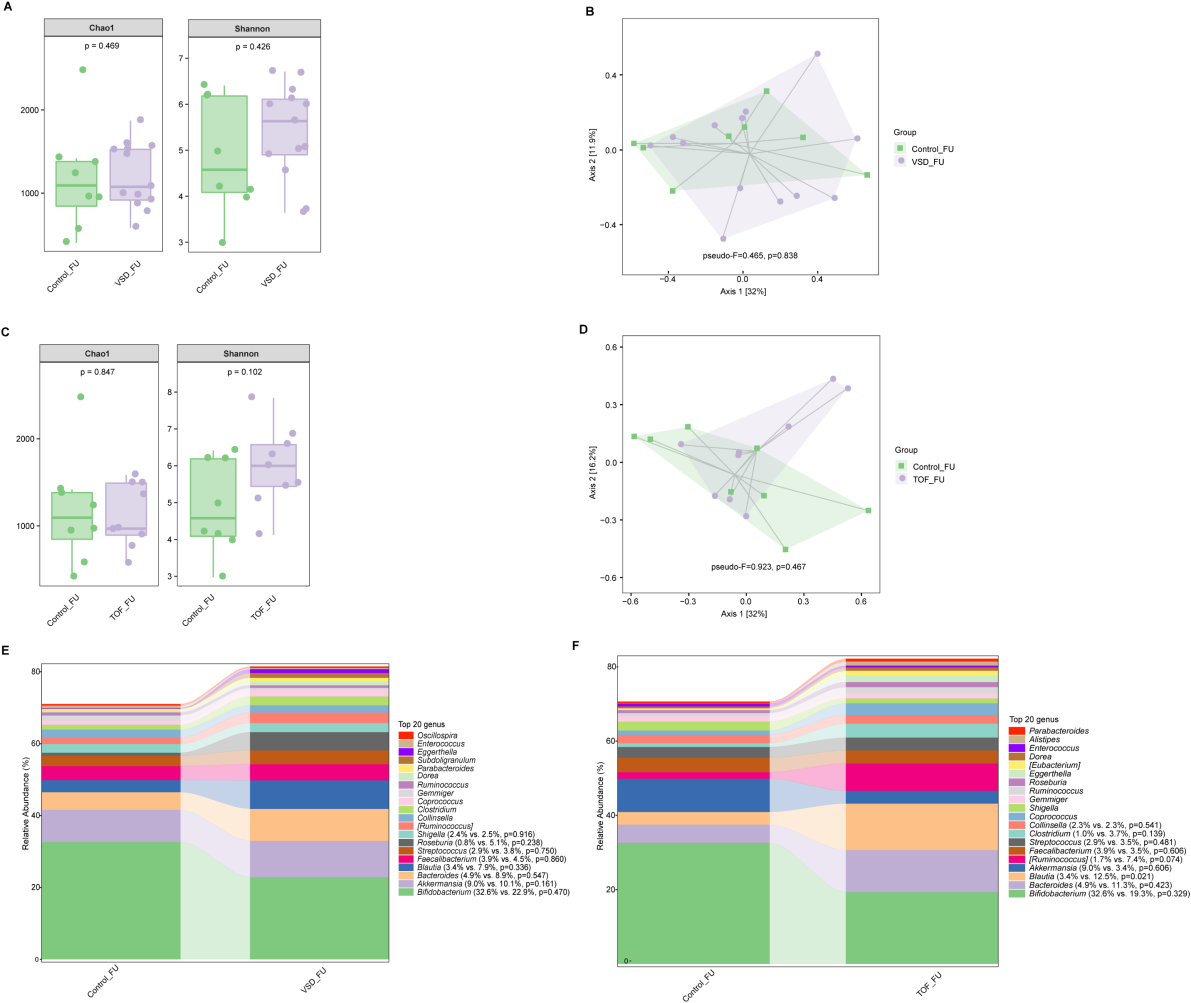
The comparisons of alpha diversity **(A)** and principal-coordinate analysis based on weighted UniFrac distance **(B)** between Control_FU and VSD_FU groups. The comparisons of alpha diversity **(C)** and principal-coordinate analysis based on weighted UniFrac distance **(D)** between Control_FU and TOF_FU groups. The top 20 relative abundances of bacteria at genus level in Control_FU and VSD_FU groups **(E)**, and in Control_FU and TOF_FU groups **(F)**. VSD_FU, one-year postoperative group with ventricular septal defect; TOF_FU, one-year postoperative group with tetralogy of Fallot; Control_FU, control group corresponding to one-year postoperative groups.

There was no significant difference in alpha diversity and beta diversity between Control_FU and VSD_FU groups (*Ps*≥ 0.426 ) (**Figures 5A, B**), nor between Control_FU and TOF_FU groups (*Ps*≥ 0.102) (**Figures 5C, D**). Compared with Control_FU group, the relative abundances of dominated genera were not significantly different in VSD_FU and TOF_FU groups respectively (*Ps*≥ 0.074 ), except for *Bluatia* being increased in TOF_FU group than in Control_FU group (*P*=0.021) (**Figures 5E, F**). 

**Figure 6:**
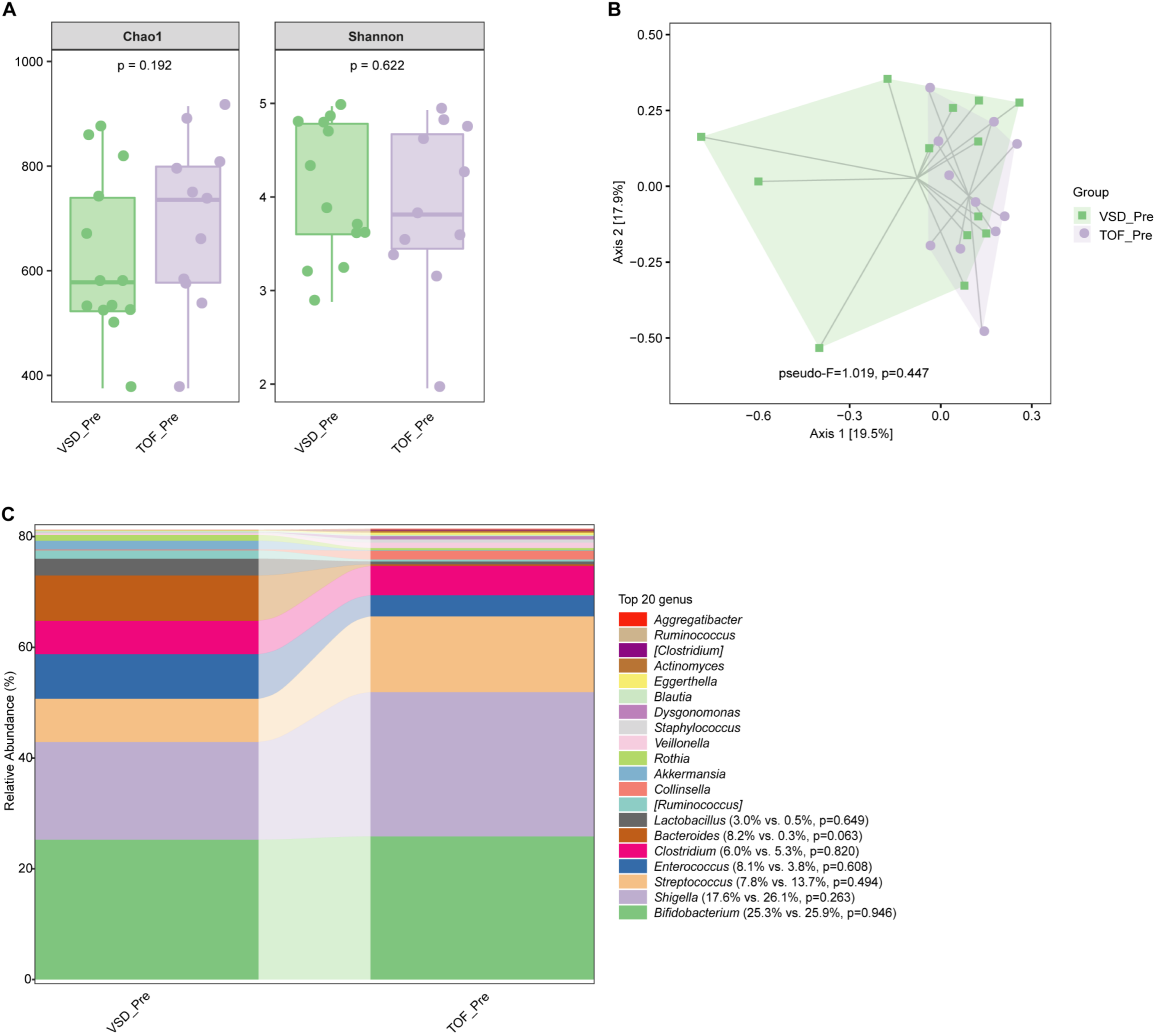
The comparisons of alpha diversity **(A)** and principal-coordinate analysis based on weighted UniFrac distance **(B)** between VSD_Pre and TOF_Pre groups, and the top 20 relative abundances of bacteria at genus level in them **(C)**. VSD_Pre, preoperative group with ventricular septal defect; TOF_Pre, preoperative group with tetralogy of Fallot.

**Figure 7:**
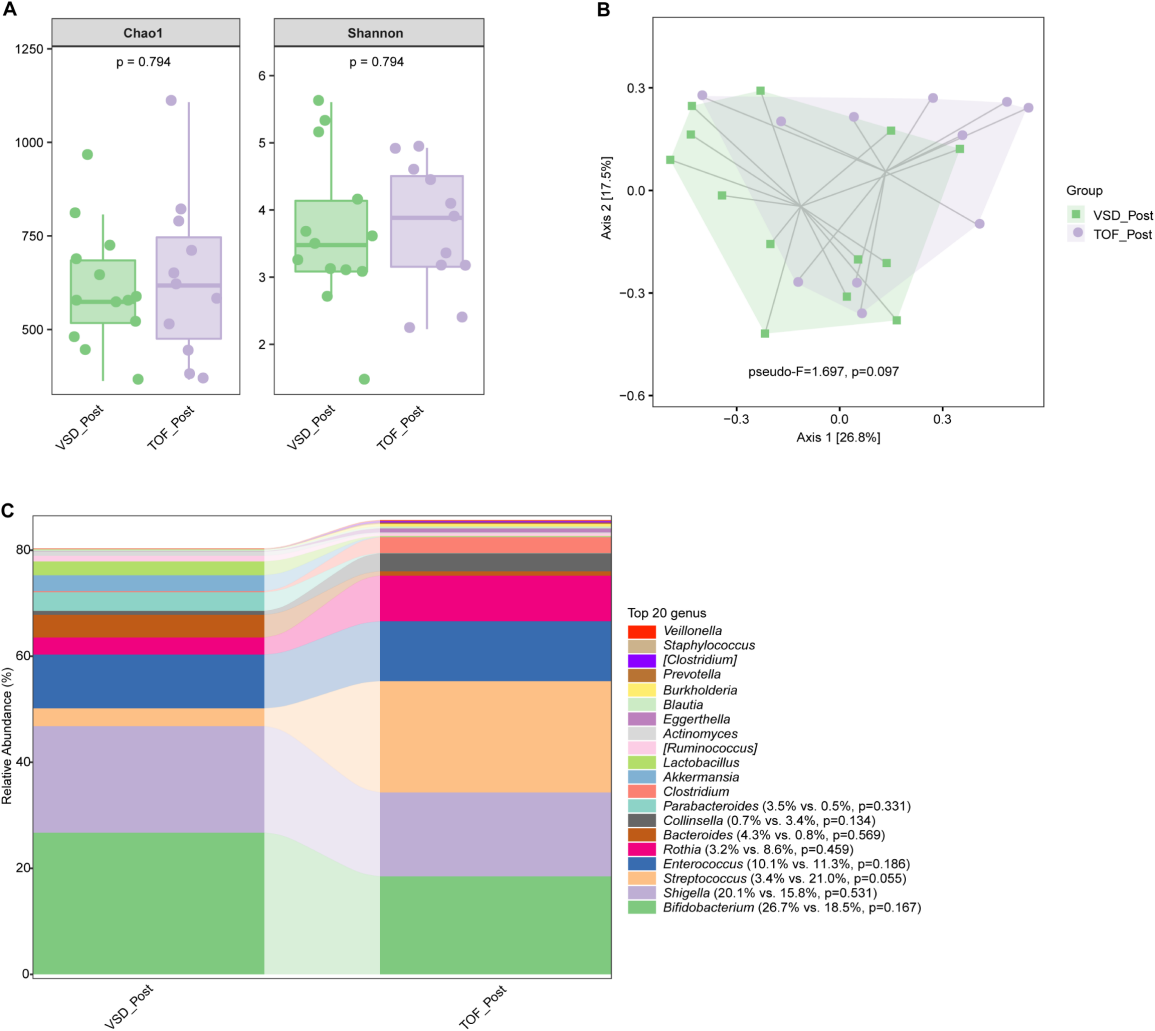
The comparisons of alpha diversity **(A)** and principal-coordinate analysis based on weighted UniFrac distance **(B)** between VSD_Post and TOF_Post groups, and the top 20 relative abundances of bacteria at genus level in them **(C)**. VSD_Post, early postoperative group with ventricular septal defect; TOF_Post, early postoperative group with tetralogy of Fallot.

**Figure 8:**
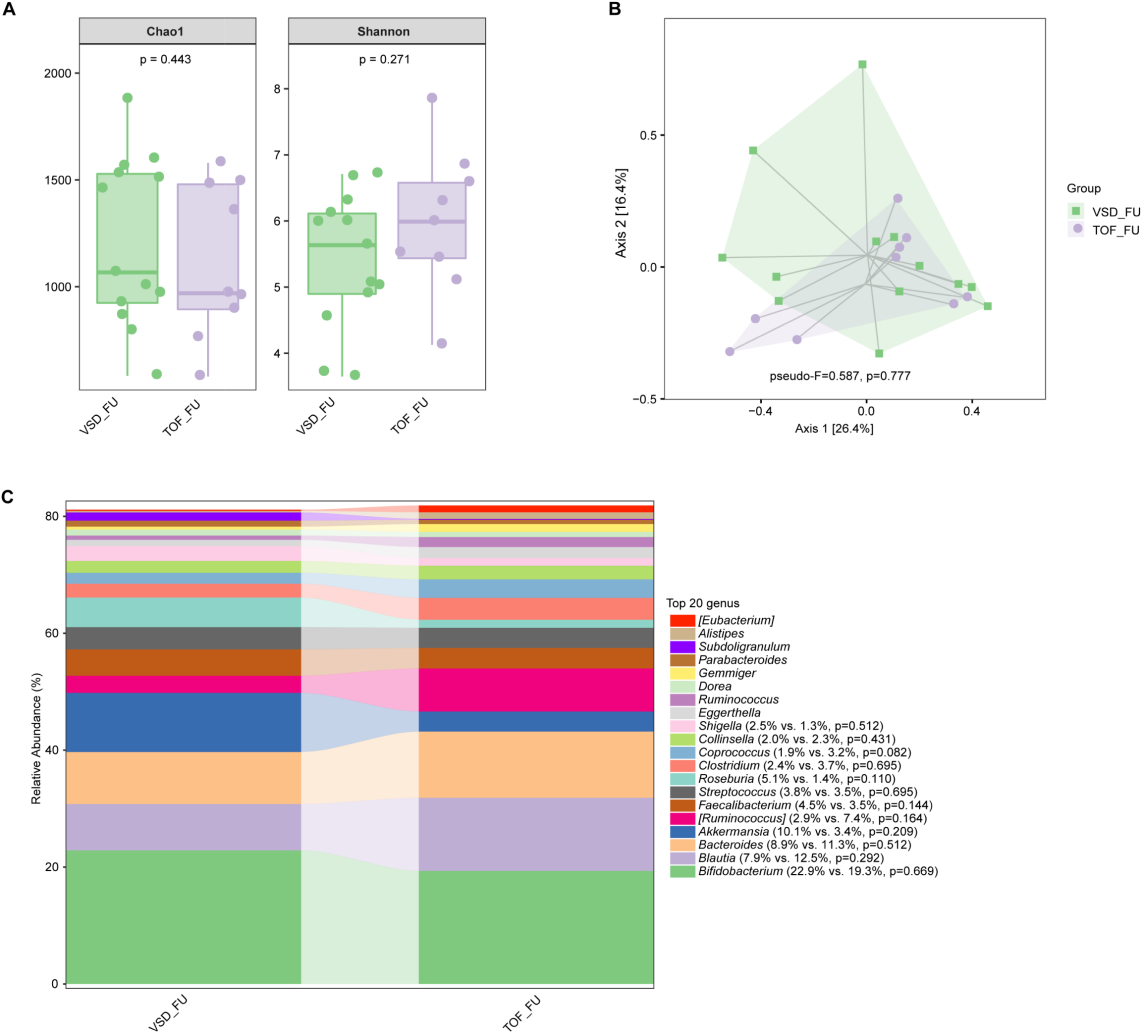
The comparisons of alpha diversity **(A)** and principal-coordinate analysis based on weighted UniFrac distance **(B)** between VSD_FU and TOF_FU groups, and the top 20 relative abundances of bacteria at genus level in them **(C)**. VSD_FU, one-year postoperative group with ventricular septal defect; TOF_FU, one-year postoperative group with tetralogy of Fallot.

No significant difference in alpha diversity, beta diversity, and the relative abundances of abundant genera were found between VSD and TOF groups at any of the 3 study periods (*Ps*≥ 0.055) (**Figures 6A–C**, **7A–C**, **8A–C**).

## Discussion

The present study demonstrated that, in both groups of children with VSD or TOF, gut microbiota dysbiosis existed preoperatively and were not disturbed significantly by CPB. One-year post-CPB, gut microbiota tended to be normal. There was no significant difference in microbial communities between VSD and TOF groups throughout the 3 periods.

Preoperatively, harmful taxa Enterobacteriaceae and *Shigella* were the characteristic bacteria in both groups of children with VSD and TOF, and the relative abundance of *Bifidobacterium* was lower compared to corresponding control groups, despite the high incidence of vaginal delivery in VSD_Pre group. Actually, vaginal-born infants presented the high relative abundances of *Bifidobacterium* (Reyman et al., 2019). Depletion of *Bifidobacterium* has been shown to induce systemic inflammation and immune imbalance (Henrick et al., 2021). A large-scale cohort study (n=7211) has demonstrated that Enterobacteriaceae was strongly associated with high all-cause mortality in gastrointestinal and respiratory events (Salosensaari et al., 2021). The underlying mechanisms for gut microbiota dysbiosis in children with CHD remain poorly understood. It has been reported that children with CHD have lost the integrity of intestinal epithelium preoperatively (Typpo et al., 2015), which may indicate intestinal inflammation (Subramanian et al., 2020). The intestinal inflammation may change gut bacteria to support the overgrowth of aerotolerant bacteria, especially Enterobacteriaceae (Lupp et al., 2007). Its expansion is considered as a common marker of gut dysbiosis (Byndloss et al., 2017).

Previous studies have largely focused on cyanotic and severe CHD. For example, patients with TOF pre-CPB presented microbial dysbiosis with changed taxonomic compositions and impaired functional profiles (Liu et al., 2022). In neonates with critical CHD, gut dysbacteriosis were shown to be related to metabolomic perturbations and involved in immune imbalance and adverse clinical outcomes (Huang et al., 2022). Only one study compared gut microbiota between cyanotic and non-cyanotic patients. It showed that gut microbiota of patients with cyanotic CHD (TOF, double outlet right ventricle, transposition of the great arteries, and pulmonary artery atresia) differed from that of non-cyanotic patients (VSD and atrial septal defect) markedly with decreased proportion of *Lactobacilli* (Xing et al., 2018). Data from our present study showed that there was no significant difference in microbial community between VSD_Pre and TOF_Pre groups. This is different from our hypothesis and might be attributable to the different types of CHD between the two studies. As described above, the previous study included more severe CHD besides TOF, which may be associated with poorer intestinal oxygen supply as a result of a combination of hypoperfusion and hypoxemia.

It is also to our surprise that our data did not show significant difference in microbial communities between pre-CPB and early post-CPB periods in both VSD and TOF groups. It is well known that CPB induces intense systemic inflammation responses (Warltier et al., 2002), ischemia-reperfusion injury (Collard and Gelman, 2001) and imbalanced oxygen transport with reduced oxygen delivery (Li et al., 2000, 2007), and that the intestinal epithelia are particularly sensitive to ischemic injury (Robinson and Mirkovitch, 1972). Deng et al. established intestinal ischemia-reperfusion model in mouse and observed that gut microbiota was substantially modified at the end of reperfusion for 2 hours (Deng et al., 2021). There might be other reasons attributable to the similar microbial communities between pre- and post-CPB patients. Gut microbiota presents the property of certain resilience that the microbial dysbiosis can recover following a short-term catastrophic perturbation, i.e., inflammation (Sommer et al., 2017). A study found a temporary pattern of bacterial changes in ischemia-reperfusion rats that the intestinal communities altered early after intestinal reperfusion and reached significant differences at 12 hours following reperfusion, then recovered toward normal pattern by 72 hours following reperfusion (Wang et al., 2013). Besides, it is known that intestinal epithelia and microbiota interact intimately (Kayama et al., 2020). Studies focusing on perioperative epithelial injury found that the plasma level of intestinal fatty acid-binding protein, an indicator of intestinal epithelial injury (Watson et al., 2020), was increased initially at rewarming and then decreased gradually within 24 to 48 hours after CPB, suggesting restoration of intestinal epithelia (Salomon et al., 2021; Pathan et al., 2011; Subramanian et al., 2020; Adamik et al., 2017). As such, the early post-CPB microbiota in our patients might have been changed to some degree but then gradually recovered to the pre-CPB pattern by the time of sampling. Nonetheless, Salomon et al. reported exacerbated gut bacteria dysbiosis in children with CHD 2 to 4 days after CPB compared to the preoperative samples (Salomon et al., 2021). This, again, might be due to more severe CHD, more complex surgery and longer duration of CPB in their study population as described above. Adamik et al. reported that the adult patients undergoing longer duration of CPB≥90 minutes had higher levels of intestinal fatty acid-binding protein and endotoxin relating with gut translocation, compared to those with duration of CPB<90 minutes (Watson et al., 2020). In our patients, TOF_Post group had a significantly longer CPB time compared to VSD_Post group [75 (84-64) vs. 115 (126-96), *P*<0.001]. However, microbial community of TOF_Post group was similar with that of VSD_Post group. This might be due partly to the similar baseline microbiota pre-CPB between the two groups.

Expectedly, our study showed that about one year after CPB, gut microbiota recovered close to normal patterns in both TOF_FU and VSD_FU groups. The relative abundances of harmful bacteria, such as *Enterococcus* and *Rothia*, were decreased, and the beneficial SCFAs-producing taxa became dominant. This indicates that the dysbacteriosis observed pre- and early post-CPB was ameliorated during this period with fairly normal hemodynamics after surgical correction of CHD.

## Limitations

Our study had a couple of limitations. First, while the same cohorts of patients with VSD and TOF were longitudinally studied before and early post-CPB, a cross-sectional design was used between early and one-year post-CPB groups, due to the relatively short study period. This might introduce some bias but unlikely influence our final results. Second, our study was conducted in a single center with small sample sizes, thus, limiting the generalizability of our results.

## Conclusions

In children with VSD or TOF, gut microbiota dysbiosis existed with the overgrowth of harmful bacteria before CPB. Overall communities were not significantly altered by CPB. About one year after CPB, microbiota was largely restored close to normal. Similar microbial communities were found between children with VSD and TOF throughout perioperative and long-term postoperative periods. Further studies are warranted to define the characteristics of gut microbiota dysbiosis in varied types of CHD in larger patient populations. Perioperative treatment targeting on gut microbiota may provide more beneficially effects on the growth and development following CPB in this vulnerable group of patients.

## Data Availability

The datasets presented in this study can be found in online repositories. The names of the repository/repositories and accession number(s) can be found in the article/supplementary material.

## References

[B1] Adamik B. Kübler A. Gozdzik A. Gozdzik W. (2017). Prolonged cardiopulmonary bypass is a risk factor for intestinal ischaemic damage and endotoxaemia. Heart Lung Circ. 26, 717–723. doi: 10.1016/j.hlc.2016.10.012 27956161

[B2] Byndloss M. X. Olsan E. E. Rivera-Chávez F. Tiffany C. R. Cevallos S. A. Lokken K. L.. (2017). Microbiota-activated PPAR-γ signaling inhibits dysbiotic Enterobacteriaceae expansion. Science 357, 570–575. doi: 10.1126/science.aam9949 28798125 PMC5642957

[B3] Carlson A. L. Xia K. Azcarate-Peril M. A. Goldman B. D. Ahn M. Styner M. A.. (2018). Infant gut microbiome associated with cognitive development. Biol. Psychiatry 83, 148–159. doi: 10.1016/j.biopsych.2017.06.021 28793975 PMC5724966

[B4] Clemente J. C. Ursell L. K. Parfrey L. W. Knight R. (2012). The impact of the gut microbiota on human health: an integrative view. Cell 148, 1258–1270. doi: 10.1016/j.cell.2012.01.035 22424233 PMC5050011

[B5] Collard C. D. Gelman S. (2001). Pathophysiology, clinical manifestations, and prevention of ischemia-reperfusion injury. Anesthesiology 94(6), 1133–8. doi: 10.1097/00000542-200106000-00030 11465607

[B6] de Muinck E. J. Trosvik P. (2018). Individuality and convergence of the infant gut microbiota during the first year of life. Nat. Commun. 9, 2233. doi: 10.1038/s41467-018-04641-7 29884786 PMC5993781

[B7] Deng F. Zhao B. C. Yang X. Lin Z. B. Sun Q. S. Wang Y. F.. (2021). The gut microbiota metabolite capsiate promotes Gpx4 expression by activating TRPV1 to inhibit intestinal ischemia reperfusion-induced ferroptosis. Gut Microbes 13, 1–21. doi: 10.1080/19490976.2021.1902719 PMC8009132 33779497

[B8] Feng D. Christensen J. T. Yetman A. T. Lindsey M. L. Singh A. B. Salomon J. D. (2021). The microbiome’s relationship with congenital heart disease: more than a gut feeling. J. Congenital Cardiol. 5, 1–11. doi: 10.1186/s40949-021-00060-4

[B9] Henrick B. M. Rodriguez L. Lakshmikanth T. Pou C. Henckel E. Arzoomand A.. (2021). Bifidobacteria-mediated immune system imprinting early in life. Cell 184, 3884–3898.e11. doi: 10.1016/j.cell.2021.05.030 34143954

[B10] Hou K. Wu Z. X. Chen X. Y. Wang J. Q. Zhang D. Xiao C.. (2022). Microbiota in health and diseases. Signal Transduct Target Ther. 7, 135. doi: 10.1038/s41392-022-00974-4 35461318 PMC9034083

[B11] Huang Y. Lu W. Zeng M. Hu X. Su Z. Liu Y.. (2022). Mapping the early life gut microbiome in neonates with critical congenital heart disease: multiomics insights and implications for host metabolic and immunological health. Microbiome 10, 245. doi: 10.1186/s40168-022-01437-2 36581858 PMC9801562

[B12] Kayama H. Okumura R. Takeda K. (2020). Interaction between the microbiota, epithelia, and immune cells in the intestine. Annu. Rev. Immunol. 38, 23–48. doi: 10.1146/annurev-immunol-070119-115104 32340570

[B13] Li J. Schulze-Neick I. Lincoln C. Shore D. Scallan M. Bush A. (2000). Oxygen consumption after cardiopulmonary bypass surgery in children: determinants and implications. J Thorac Cardiovasc Surg. 119 (3), 525–33. doi: 10.1016/s0022-5223(00)70132-2 10694613

[B14] Li J. Zhang G. McCrindle B. W. Holtby H. Humpl T. Cai S. (2007). Profiles of hemodynamics and oxygen transport derived by using continuous measured oxygen consumption after the Norwood procedure. J Thorac Cardiovasc Surg. 133 (2), 4418. doi: 10.1016/j.jtcvs.2006.09.033 17258581

[B15] Liu X. Lu S. Shao Y. Zhang D. Tu J. Chen J. (2022). Disorders of gut microbiota in children with Tetralogy of Fallot. Transl. Pediatr. 11, 385–395. doi: 10.21037/tp-22-33 35378966 PMC8976677

[B16] Lupp C. Robertson M. L. Wickham M. E. Sekirov I. Champion O. L. Gaynor E. C.. (2007). Host-mediated inflammation disrupts the intestinal microbiota and promotes the overgrowth of Enterobacteriaceae. Cell Host Microbe 2, 204. doi: 10.1016/j.chom.2007.08.002 18030708

[B17] Pathan N. Burmester M. Adamovic T. Berk M. Ng K. W. Betts H.. (2011). Intestinal injury and endotoxemia in children undergoing surgery for congenital heart disease. Am. J. Respir. Crit. Care Med. 184, 1261–1269. doi: 10.1164/rccm.201104-0715OC 21868501

[B18] Patterson G. T. Osorio E. Y. Peniche A. Dann S. M. Cordova E. Preidis G. A.. (2022). Pathologic inflammation in malnutrition is driven by proinflammatory intestinal microbiota, large intestine barrier dysfunction, and translocation of bacterial lipopolysaccharide. Front. Immunol. 13. doi: 10.3389/fimmu.2022.846155 PMC9204284 35720380

[B19] Reyman M. van Houten M. A. van Baarle D. Bosch A. Man W. H. Chu M.. (2019). Impact of delivery mode-associated gut microbiota dynamics on health in the first year of life. Nat. Commun. 10, 4997. doi: 10.1038/s41467-019-13014-7 31676793 PMC6825150

[B20] Robinson J. W. Mirkovitch V. (1972). The recovery of function and microcirculation in small intestinal loops following ischaemia. Gut. 13(10), 784–9. doi: 10.1136/gut.13.10.784 PMC1412475 4263959

[B21] Roswall J. Olsson L. M. Kovatcheva-Datchary P. Nilsson S. Tremaroli V. Simon M. C.. (2021). Developmental trajectory of the healthy human gut microbiota during the first 5 years of life. Cell Host Microbe 29, 765–776.e3. doi: 10.1016/j.chom.2021.02.021 33794185

[B22] Salomon J. Ericsson A. Price A. Manithody C. Murry D. J. Chhonker Y. S.. (2021). Dysbiosis and intestinal barrier dysfunction in pediatric congenital heart disease is exacerbated following cardiopulmonary bypass. JACC Basic Transl. Sci. 6, 311–327. doi: 10.1016/j.jacbts.2020.12.012 33997519 PMC8093480

[B23] Salosensaari A. Laitinen V. Havulinna A. S. Meric G. Cheng S. Perola M.. (2021). Taxonomic signatures of cause-specific mortality risk in human gut microbiome. Nat. Commun. 12, 2671. doi: 10.1038/s41467-021-22962-y 33976176 PMC8113604

[B24] Shi H. Hu C. Zhang L. Tong M. Li L. Cui Y. (2021). Early growth trajectory of infants with simple congenital heart disease and complex congenital heart disease undergoing cardiac repair: A prospective cohort study in China. JPEN J. Parenter Enteral Nutr. 45, 1181–1191. doi: 10.1002/jpen.2017 32914899

[B25] Sommer F. Anderson J. M. Bharti R. Raes J. Rosenstiel P. (2017). The resilience of the intestinal microbiota influences health and disease. Nat. Rev. Microbiol. 15, 630–638. doi: 10.1038/nrmicro.2017.58 28626231

[B26] Subramanian S. Geng H. Tan X. D. (2020). Cell death of intestinal epithelial cells in intestinal diseases. Sheng Li Xue Bao 72, 308–324. 32572429 PMC7755516

[B27] Tsukuda N. Yahagi K. Hara T. Watanabe Y. Matsumoto H. Mori H.. (2021). Key bacterial taxa and metabolic pathways affecting gut short-chain fatty acid profiles in early life. Isme J. 15, 2574–2590. doi: 10.1038/s41396-021-00937-7 33723382 PMC8397723

[B28] Typpo K. V. Larmonier C. B. Deschenes J. Redford D. Kiela P. R. Ghishan F. K. (2015). Clinical characteristics associated with postoperative intestinal epithelial barrier dysfunction in children with congenital heart disease. Pediatr. Crit. Care Med. 16, 37–44. doi: 10.1097/pcc.0000000000000256 25162512 PMC4286428

[B29] Wang F. Li Q. He Q. Geng Y. Tang C. Wang C.. (2013). Temporal variations of the ileal microbiota in intestinal ischemia and reperfusion. Shock 39, 96–103. doi: 10.1097/SHK.0b013e318279265f 23247126

[B30] Warltier D. C. Laffey J. G. Boylan J. F. Cheng D. C. H. (2002). The systemic inflammatory response to cardiac surgery. Anesthesiology 97, 215–252. doi: 10.1097/00000542-200207000-00030 12131125

[B31] Watson J. D. Urban T. T. Tong S. S. Zenge J. Khailova L. Wischmeyer P. E.. (2020). Immediate post-operative enterocyte injury, as determined by increased circulating intestinal fatty acid binding protein, is associated with subsequent development of necrotizing enterocolitis after infant cardiothoracic surgery. Front. Pediatr. 8. doi: 10.3389/fped.2020.00267 PMC7267022 32537446

[B32] Wijeyesekera A. Wagner J. De Goffau M. Thurston S. Rodrigues Sabino A. Zaher S.. (2019). Multi-compartment profiling of bacterial and host metabolites identifies intestinal dysbiosis and its functional consequences in the critically ill child. Crit. Care Med. 47, e727–e734. doi: 10.1097/ccm.0000000000003841 31169619 PMC6699985

[B33] Xing J. Ying Y. Mao C. Liu Y. Wang T. Zhao Q.. (2018). Hypoxia induces senescence of bone marrow mesenchymal stem cells via altered gut microbiota. Nat. Commun. 9, 2020. doi: 10.1038/s41467-018-04453-9 29789585 PMC5964076

[B34] Yassour M. Vatanen T. Siljander H. Hämäläinen A. M. Härkönen T. Ryhänen S. J.. (2016). Natural history of the infant gut microbiome and impact of antibiotic treatment on bacterial strain diversity and stability. Sci. Transl. Med. 8, 343ra81. doi: 10.1126/scitranslmed.aad0917 PMC5032909 27306663

[B35] Zhang Q. L. Chen X. H. Zhou S. J. Lei Y. Q. Huang J. S. Chen Q.. (2023). Relationship between disorders of the intestinal microbiota and heart failure in infants with congenital heart disease. Front. Cell Infect. Microbiol. 13. doi: 10.3389/fcimb.2023.1152349 PMC10036851 36968106

